# *Staphylococcus aureus* injection drug use-associated bloodstream infections are propagated by community outbreaks of diverse lineages

**DOI:** 10.1038/s43856-021-00053-9

**Published:** 2021-11-30

**Authors:** Laura R. Marks, Juan J. Calix, John A. Wildenthal, Meghan A. Wallace, Sanjam S. Sawhney, Eric M. Ransom, Michael J. Durkin, Jeffrey P. Henderson, Carey-Ann D. Burnham, Gautam Dantas

**Affiliations:** 1 Division of Infectious Diseases, Washington University School of Medicine, St. Louis, MO USA; 2 The Edison Family Center for Genome Sciences and Systems Biology, Washington University School of Medicine, St. Louis, MO USA; 3 Department of Pathology and Immunology, Washington University School of Medicine, St. Louis, MO USA; 4 Department of Molecular Microbiology, Washington University School of Medicine, St. Louis, MO USA; 5 Department of Biomedical Engineering, Washington University in St. Louis, St. Louis, MO USA

**Keywords:** Infectious-disease epidemiology, Bacterial infection

## Abstract

**Background:**

The ongoing injection drug use (IDU) crisis in the United States has been complicated by an emerging epidemic of *Staphylococcus aureus* IDU-associated bloodstream infections (IDU-BSI).

**Methods:**

We performed a case-control study comparing *S. aureus* IDU-BSI and non-IDU BSI cases identified in a large US Midwestern academic medical center between Jan 1, 2016 and Dec 21, 2019. We obtained the whole-genome sequences of 154 *S. aureus* IDU-BSI and 91 *S. aureus* non-IDU BSI cases, which were matched with clinical data. We performed phylogenetic and comparative genomic analyses to investigate clonal expansion of lineages and molecular features characteristic of IDU-BSI isolates.

**Results:**

Here we show that patients with IDU-BSI experience longer durations of bacteremia and have lower medical therapy completion rates. In phylogenetic analyses, 45/154 and 1/91 contemporaneous IDU-BSI and non-IDU BSI staphylococcal isolates, respectively, group into multiple, unique clonal clusters, revealing that pathogen community transmission distinctively spurs IDU-BSI. Lastly, multiple *S. aureus* lineages deficient in canonical virulence genes are overrepresented among IDU-BSI, which may contribute to the distinguishable clinical presentation of IDU-BSI cases.

**Conclusions:**

We identify clonal expansion of multiple *S. aureus* lineages among IDU-BSI isolates, but not non-IDU BSI isolates, in a community with limited access to needle exchange facilities. In the setting of expanding numbers of staphylococcal IDU-BSI cases consideration should be given to treating IDU-associated invasive staphylococcal infections as a communicable disease.

## Abstract (web summary)

Marks and Calix et al. perform a case-control genomic epidemiology study comparing *Staphylococcus aureus* isolates from intravenous drug use-associated bloodstream infections with those from other bloodstream infections. The authors show there is clonal expansion of lineages in intravenous drug use-associated bloodstream infections suggesting person-to-person transmission of S. aureus.

## Plain language summary

Persons who inject drugs are at increased risk of developing a bloodstream infection caused by the bacterium *Staphylococcus aureus*. To investigate whether this risk is due to transmission of the bacterium within this community, we compared the complete set of genes (genome) of *S. aureus* isolated from people with bloodstream infections who do and do not inject drugs. *S. aureus* causing bloodstream infections in persons who inject drugs were much more likely to belong to one of multiple networks of very closely related subtypes, demonstrating that in some communities the bacteria causing this type of infection can likely be directly transmitted from person to person through high-risk injection drug use practices, such as sharing needles. Therefore, invasive infections in persons who inject drugs can spread like communicable diseases and this can inform future policy on how to prevent them.

## Introduction

The injection drug use (IDU) crisis in the United States (US) is complicated by emerging syndemics of infectious diseases among people who inject drugs (PWID). Since 2015 the Centers for Disease Control and Prevention and health departments across the US have identified human immunodeficiency virus (HIV) and viral hepatitis outbreaks attributed to bloodborne transmission within IDU networks^1,2^. Co-epidemics of invasive bacterial infections among PWID have also been identified^3,4^. *Staphylococcus aureus* is the most common pathogen causing these invasive infections, which are often associated with staphylococcal bloodstream infection (BSI)^5,6^. PWID are approximately 16.3 times more likely than peers to develop invasive staphylococcal infections, with one in every ten invasive staphylococcal infections in the US now related to IDU^5^. Efforts against invasive staphylococcal infections in general are complicated by the existence of multiple disease subtypes, including central line-associated BSI (CLABSI), endocarditis, osteomyelitis, septic arthritis, epidural abscess. Each disease manifestation necessitates preventative and therapeutic strategies tailored to characteristic epidemiology, pathobiology, and host risk factors^7,8^. However, the epidemiology and transmission of IDU-associated *S. aureus* invasive infections is poorly defined, as few studies have investigated it as a separate disease entity.

Staphylococcal IDU-associated bloodstream infections (IDU-BSI) may be spurred by factors characteristic of PWID, but absent among individuals with conventional forms of BSI (cBSI). For example, high rates of *S. aureus* contamination of cookers and filters used in preparation of controlled-release opioids for IDU have been observed^9^. Alternatively, it is speculated that person-to-person transmission through contaminated needles contributes to IDU-associated infections. Early reports on an IDU-associated outbreak in Detroit, US identified being unhoused and shared injection equipment as risk factors^10^, and phage typing and antibiotic susceptibility data identified common *S. aureus* lineages shared by many of these cases^11^. Subsequent reports further argued a link between IDU and transmission of methicillin-resistant *S. aureus* (MRSA) lineages among PWID^12–14^. However, these studies lacked comparator groups and/or employed low-resolution molecular methods and small cohort sizes, which limits the interpretation of their findings. As community IDU-associated transmission represents an attractive target for BSI prevention, the existence and impact of person-to-person transmission of pathogenic strains among PWID must be clearly established.

Other findings support distinguishing IDU-BSI from cBSI. IDU-associated staphylococcal infections have been associated with prolonged bacteremia duration and infectious sequelae such as endocarditis, possibly due to challenges faced by PWID in completing the standard-of-care, multiweek treatment regimens prescribed by guidelines^5^. Despite these poor prognostic indicators, emerging evidence suggests that, compared to cBSI, IDU-BSI exhibits comparable to lower mortality rates^15–20^. A mix of host and pathogen factors likely influence these discrepant observations. However, the paucity of studies comparing IDU-BSI to non-IDU staphylococcal BSI has resulted in a critical knowledge gap in our understanding of unique factors governing IDU-associated invasive disease.

We hypothesize that features unique to PWID mediate biological differences between *S. aureus* IDU-BSI and cBSI. Firstly, shared behaviors and socioeconomic conditions associated with IDU may predispose to characteristic clonal expansion of *S. aureus* pathogenic strains among PWID, raising the possibility that IDU-BSI is a communicable disease. Secondly, IDU practices predispose to direct inoculation of bacteria into the bloodstream. This bypass of major immunological barriers may obviate the role of microbial factors that mediate early stages of infection in cBSI and permit a wider diversity of otherwise-less virulent *S. aureus* strains to cause invasive disease in PWID. To investigate this, we performed extensive comparative genomic analysis of clinical isolates in a case-control study of *S. aureus* BSI occurring in a large U.S. Midwest medical center over a 4-year period. We identified clonal expansion of multiple *S. aureus* lineages among IDU-BSI isolates, but not non-IDU BSI isolates, in a community with limited access to needle exchange facilities.

## Methods

### Setting and case definitions

This study was approved by the Institutional Review Board of Washington University in St. Louis (IRB# 201804183, 201907187, 201911072, 202007171). A waiver of informed consent was granted by the Washington University IRB for isolate collection as all S. aureus specimens were obtained during routine clinical care and saved for quality improvement purposes. A waiver of informed consent for data abstraction from the chart was issued as data were pre-existing in the chart and many patients were already deceased or would otherwise have been unable to be contacted. This study was performed at Barnes-Jewish Hospital (BJH), a 1250-bed, academic, tertiary care center serving the greater metropolitan area of St. Louis, Missouri and surrounding areas. Through electronic health record (EHR) review, we retrospectively identified cases between 1/2016–1/2019 associated with *S. aureus* recovered from blood cultures or heart valve surgical specimens during routine medical care, as described previously^21^. We also included cases prospectively identified between 1/2019-12/2019 as part of a local quality improvement initiative and a CDC Developing Healthcare Safety Research Contract. Only the first documentation of infection per patient was included in our analysis, and the date of the first *S. aureus* isolate per patient (“index date”) was identified for each case. Cases were manually reviewed by an infectious diseases physician (L.R.M.) and classified as “IDU-BSI” (defined as IDU directly preceding index date) or “non-IDU” (cases lacking history of any mode of substance use), as described previously^21^. Patient demographics, drug use history, clinical course, and clinical microbiological data were obtained from EHR. Duration of bacteremia was defined as the number of calendar days between index date and date of last positive surveillance blood culture^22^. Prolonged bacteremia was defined as duration of culture-proven bacteremia ≥5 days.

As a composite comorbidity indicator, we calculated each patient’s Elixhauser Comorbidity Index by screening for 31 comorbidity diagnoses among ICD-10 codes documented prior to the index date^23^. To compare survival distributions for primary endpoint of 1-year mortality (measured from date of hospital admission to account for in-hospital deaths), we examined univariate associations with potential demographic, infection type and medical comorbidity confounders. Variables with *p* < 0.1 in univariate analyses were integrated into a multivariate cox proportional hazards model. “Drug abuse” was excluded from Elixhauser Comorbidities as this risk factor was already captured under ‘injection drug use origin’. Covariates were assessed using backwards stepwise regression, for violation of the proportional hazards assumption and assessed using log-negative-log survival plots. Hazard ratios (HRs) and 95% confidence intervals were calculated. Descriptive statistics were calculated using SPSS v26 (Chicago, IL). Figures were created using GraphPad Prism 9 (San Diego, CA).

### Identification, susceptibility testing and banking of *S. aureus* isolates

**Fig. 1:**
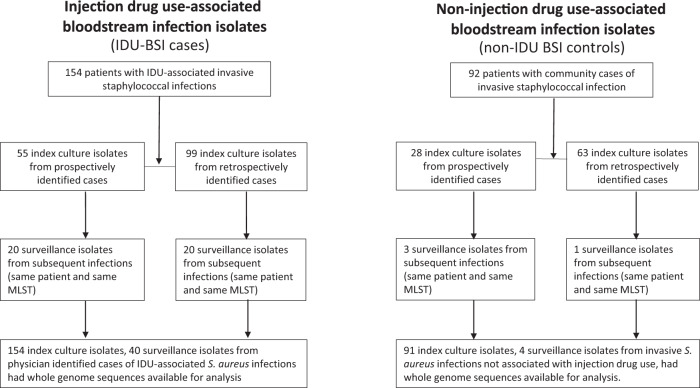
Isolate Classification. Flowchart and classification of study patients based on injection drug use history, study period, and subsequent episode of recurrent *S*. *aureus* infection. Abbreviations: BSI, bloodstream infection; IDU, injection drug use; IDU-BSI, injection drug use associated bloodstream infection; MLST, multilocus sequence type.

*S. aureus* clinical isolates were cultured and analyzed in the BJH clinical microbiology laboratory as part of the routine medical care. All isolates recovered from BSI are routinely stored by the BJH clinical laboratory at −80 °C in skim milk until recovered for analysis. The identification of banked isolates was done using VITEK MS MALDI-TOF MS v2.3.3. Methicillin resistance was determined using PBP2a testing (Alere), and, for isolates from blood cultures, *mecA* detection using the Verigene BC-GP assay (Luminex), in addition to cefoxitin disk diffusion. Susceptibility testing for cefoxitin (FOX), clindamycin (CLI), doxycycline (DOX), erythromycin (ERY), and trimethoprim-sulfamethoxazole (SXT) was performed by the disk diffusion method according to CLSI guidelines^24^. Isolates were classified as “index” (the first isolate obtained per patient) or “surveillance” (isolates identified subsequent to index isolates). Isolates were cross-referenced with medical records from the above case analysis and classified as IDU-BSI and non-IDU BSI cases, respectively (Fig. 1). IDU-BSI isolates were from IDU-BSI cases identified above. Non-IDU BSI isolates were selected among cases occurring during the study period in patients with no history of drug use, and matched to IDU-BSI isolates according to isolation date and patient residency (rural versus urban zip code). For secondary analyses, non-IDU BSI isolates chosen for WGS were subclassed into “CLABSI” (defined as cases in which central line venous access present at index date) or “cBSI” (defined as BSI not associated with IDU or central lines access).

### Whole-genome sequencing (WGS) and genomic analysis

Illumina sequencing was performed on genomic DNA extracted from a subset of clinical isolates (Fig. 1). Genome assembly and analysis, including core genome alignment, multilocus sequence typing (MLST), and identification of antimicrobial resistance and virulence factor genes, were performed based on well-established processing pipelines^25^. Isolates were propagated on sheep’s blood agar, and *S. aureus* isolate genomic DNA was extracted with the Qiagen Bacteremia Kit (Qiagen, Germantown, MD, USA) according to the manufacturer’s instructions. A total of 5 ng/uL of DNA was used as input for Illumina sequencing libraries with the Nextera kit (Illumina, San Diego, CA, USA). Pooled libraries were sequenced on a NextSeq HighOutput platform (Illumina) to obtain 2 × 150 bp reads. Following demultiplexing by barcode, reads had adapters removed with Trimmomatic v0.38^26^. Reads were then assembled into draft genomes using de-novo assembler Unicycler v0.4.7^27^. Scaffolds.fasta files were used for downstream analysis. Assembly statistics was quantified using QUAST v4.5^28^. Whole-genome sequence read files are uploaded to NCBI under BioProjects PRJNA694991 and PRJNA695316.

Species identity of each genome was confirmed using the ANIm method from pyANI v0.2.7^29^. ANIm ≥96% compared to the *S. aureus* reference genome (strain NCTC 8325, GCF_000013425.1) was used as the species cutoff. In silico screen for antibiotic resistance genes and multilocus sequence typing (MLST) was performed with ResFinder v4.0^30^ and MLST-check^31^, respectively. Prokka v1.13.7^32^ was run on scaffold files to identify open reading frames >500 bp in length. Scaffolds were screened for the presence of genes of interest with BLAST using the following parameters [−evalue 1e − 10 -perc_identity 90 -gapopen 5 -gapextend 5] and a cutoff of >90% amino acid identity. Screened genes were selected a priori according to their predicted role in disease, based on literature review^33,34^ and are listed in Supplementary Data 1.

### Phylogenetic and clonal analysis

For phylogenetic analysis, the gff files produced by Prokka were used to construct a core genome alignment with Roary v3.13^35^. The alignment was used to generate a maximum-likelihood tree with raxML v8.2.11^36^, and visualized with iTOL^37^. For clonality analysis, Snp-sites v2.4.0^38^ was used to remove indels and create multiFASTA alignment containing the single nucleotide polymorphism (SNP) sites for each core genome. Pairwise SNP counts between isolates were calculated and plotted. SNP distances of isolates repeatedly obtained from the same patient were used as reference, to empirically determine SNP distance cutoffs indicating clonal transmission. SNP distances between surveillance isolates obtained from the same patient served as reference to empirically determine a cutoff to define a clonal relationship between isolates obtained from different individuals. Interactions that met the cutoff were visualized using Cytoscape v4.0^39^. Source data are available in Supplementary Data 2.

### Statistics and reproducibility

Unless otherwise stated, comparisons of categorical data were performed by Fisher’s exact test, while comparisons of continuous variable were tested using the Mann-Whitney U test. All tests were two‐tailed, and statistical significance was defined as *p* ≤ 0.05 or a 95% confidence interval (CI) excluding 1.00 for an odds ratio (OR). Statistical analyses were performed on JMP Pro software (v15) or R software^40^.

## Results

### Clinical characteristics of IDU-BSI

**Fig. 2:**
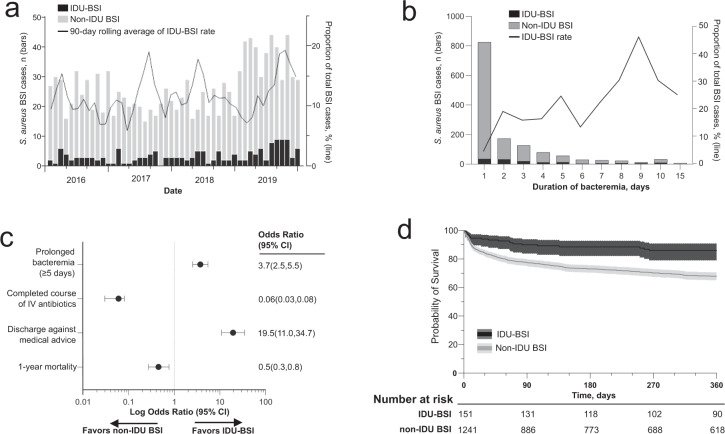
Comparing the clinical traits and IDU-BSI and non-IDU BSI. **a** Number of unique patients with *S. aureus* IDU-BSI and non-IDU BSI (bars), and 90-day rolling average proportion of total BSI cases that are IDU-associated (line) identified in BJH from 1/1/2016 to 12/31/2019. **b** Duration in days of *S. aureus* bacteremia among adults stratified by IDU-BSI and non-IDU BSI. **c** Among BSI cases, AMA discharges and prolonged bacteremia (≥5 days) was more common among IDU-BSI with lower rates of completion of standard of care intravenous (IV) antibiotics but did not translate into higher 1 year mortality rates. **d** Kaplan–Meier survival curves comparing *S. aureus* IDU-BSI and non-IDU BSI with number at risk below the graph.

We identified 173 and 1261 hospitalizations for *S. aureus* IDU-BSI and non-IDU cBSI, respectively, from January 2016 through December 2019. The proportion of cases associated with IDU increased from 9.1% in 2016 to 13.4% in 2019, with 12.1% of BSI cases being IDU-BSI during the study period (Fig. 2a). However, IDU-BSI composed 25.7% of cases of prolonged bacteremia (i.e., culture-proven bacteremia lasting ≥5 days), including 46.1% of cases with bacteremia lasting 9 days (Fig. 2b). Supplemental Table 1 summarizes patient characteristics by BSI type. IDU-BSI patients were younger, and more likely to have infective endocarditis (*p* < 0.001), while non-IDU BSI cases were more likely to have CLABSI (*p* = 0.004). Though the rate of some comorbidities differed between groups, there was no significant difference in the mean Elixhauser Comorbidities Index (*p* = 0.565, Supplementary Table 1). Compared to non-IDU BSI cases, IDU-BSI cases were more likely to experience prolonged bacteremia (*p* < 0.001) and to leave the hospital against medical advice (AMA) (*p* < 0.001), while being less likely to complete a full course of intravenous antibiotic therapy (*p* < 0.001) (Fig. 2c). Despite this, IDU-BSI cases exhibited decreased 30-day, 90-day and 1-year unadjusted mortality compared to non-IDU BSI counterparts (*p* < 0.001 for all comparisons) (Fig. 2c, d). 1-year mortality remained lower among IDU-BSI patients after adjusting for sex, age, and presence of clinically relevant Elixhauser Comorbidities (aHR 0.78; 95% CI 0.62–0.98, *p* 0.021, Supplementary Table 2).

**Table 1:** Demographics of index isolates included for whole-genome sequencing.

	Non-IDU patients, *N* = 91	PWID, *N* = 154	*P*
*Demographics*			
Age (mean, SD)	58 ± 14	38 ± 10	<0.001
Female	34 (37.4%)	77 (50%)	0.045
Homeless	0 (0.0%)	12 (7.8%)	<0.001
Discharged AMA	0 (0.0%)	43 (27.9%)	<0.001
Substance use patterns			
Opioid use (fentanyl or heroin)	0 (0.0%)	142 (92.2%)	<0.001
Methamphetamine use	0 (0.0%)	48 (31.1%)	<0.001
*Comorbidities*			
Hepatitis B virus infection	0 (0.0%)	6 (3.9%)	0.017
Hepatitis C virus infection	1 (1.1%)	95 (61.7%)	<0.001
HIV Infection	0 (0.0 %)	10 (6.5%)	0.002
Elixhauser comorbidities (mean, SD)	9.0 (3.4)	8.6 (3.9)	0.484
*Clinical syndromes caused by Isolate*			
Infective endocarditis	21 (23.0%)	99 (64.3%)	<0.001
Osteomyelitis	17 (18.6%)	33 (21.4%)	0.576
Septic arthritis	10 (10.9%)	27 (17.5%)	0.149
Necrotizing skin and soft tissue infection	6 (6.6%)	22 (14.2%)	0.054
Isolated bacteremia	46 (50.5%)	15 (9.7%)	<0.001
*S. aureus infection characteristics*			
Hospital Day of *S. aureus* Isolation for WGS (mean day, SE)	1 ± 0.2	1 ± 0.3	0.641
Duration of bacteremia (mean days, SE)	3 ± 0.3	4 ± 0.3	0.122
Central line associated bacteremia	38 (41.8%)	1 (0.6%)	<0.001
*Outcomes*			
1 year Mortality	27 (29.7%)	22 (14.3%)	0.082

*AMA* against medical advice, *HIV* human immunodeficiency virus, *IDU* injection drug use, *PWID* person who injects drugs, *SD* standard deviation, *SE* standard error, *WGS* whole-genome sequencing.

### Clinical characteristics of cases included in WGS cohort

We obtained genome sequences from 289*S. aureus* isolates from patients with *S. aureus* endovascular infections. These included 245 index and 44 surveillance isolates (Supplementary Table 1). The demographic and clinical characteristics of patients with cases associated with index IDU and non-IDU isolates chosen for WGS (*n* = 154 and 91, respectively), are compared in Table 1. Sequenced *S. aureus* IDU-BSI strains occurred among younger patients who were more likely to experience homelessness (*p* < 0.001), and to be co-infected with hepatitis B (HBV) (*p* = 0.017), hepatitis C (HCV) (*p* < 0.001), or HIV (*p* = 0.002). IDU-BSI cases were more likely to be associated with infective endocarditis (*p* < 0.001), and less likely to be associated with the presence of a central line at diagnosis or to present without metastatic sites of infection (“isolated bacteremia”) (*p* < 0.001). There was no significant difference in the occurrence of osteomyelitis, necrotizing fasciitis, or septic arthritis. The investigated characteristics of IDU-BSI and non-IDU BSI cases included in WGS analysis, were comparable to those of corresponding subgroups in the total BJH BSI cohort (Supplementary Table 1).

### Phylogenetic analysis of BSI isolates

**Fig. 3:**
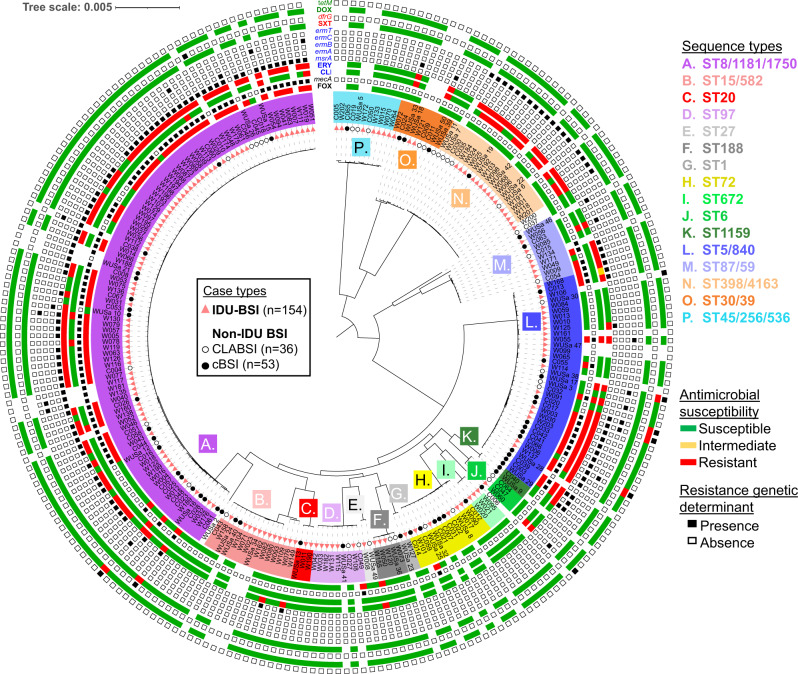
Phylogeny of BJH *S. aureus* BSI isolates. Maximum-likelihood phylogenetic tree is based on 86493 core genome SNPs and rooted to midpoint. Black-filled circles, unfilled circles and red triangles on branch tips denote cBSI, CLABSI and IDU-BSI isolates, respectively. Isolate names are highlighted with colors denoting the multilocus sequence type (ST) identity of each isolate. Single and double-locus variant STs that exclusively share a common ancestor, are grouped in 16 ST groups. Outer rings denote an isolate’s antibiotic susceptibility according to electronic medical records (multicolored strip) and the presence/absence of attributable genetic determinants according to WGS (adjacent squares). Antibiotic and gene name labels are color coded according to pertinent antibiotic class. FOX, cefoxitin; CLI, clindamycin; ERY, erythromycin; SXT, trimethoprim/sulfamethoxazole; DOX, doxycycline. Figure metadata included in Supplementary Data 3.

BSI isolate genomes represented 26 different multilocus sequence types (ST), which included 10 ST groups (i.e., groups of single-locus or double-locus MLST variants sharing a common ancestor) containing at least five index isolates (Fig. 3 and Supplementary Table 3). The distribution of these ST groups between IDU-BSI and non-IDU BSI index isolates was not significantly different (*p* = 0.081, by Fisher exact test). The MRSA-associated ST groups ST8/1181/1750 and ST5/840, composed 67.1% and 25.2% of index isolates, respectively. The most common non-MRSA ST groups, ST398/4163 and ST15/582, composed 14.0% and 10.5% of index isolates, respectively.

### Identification and characterization of staphylococcal IDU-BSI transmission clusters

**Fig. 4:**
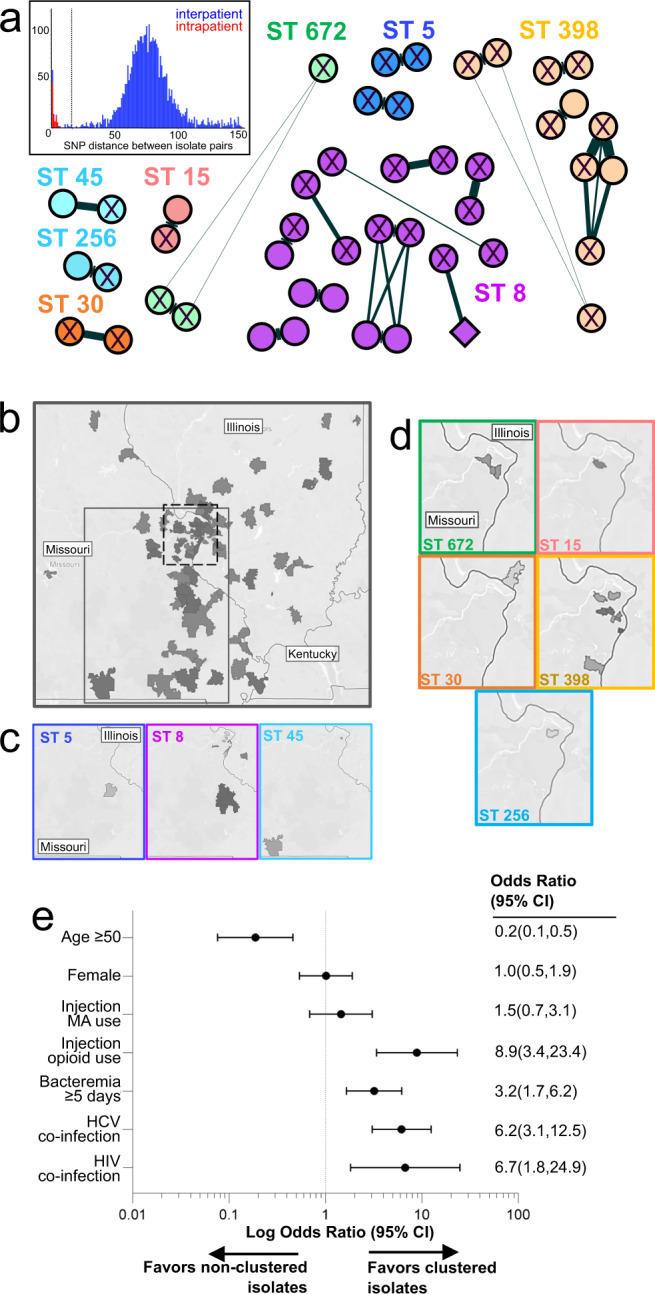
Clonal outbreaks of diverse lineages among persons who inject drugs. **a** Networks of isolates with ≤15 core genome SNPs. Each node represents one of 46 index isolates, and are color coded by MLST, shaped according to whether associated with IDU-BSI (circle) or non-IDU BSI (diamond), and labeled according to HCV serostatus (‘X’ denotes HCV co-infection). Edge length and width represent SNP distance between nodes. *Inset*, histogram depicting collective core genome SNP distances between isolate pairs obtained from separate patients (“interpatient”, blue) or from the same patient (“intrapatient”, red). For clarity only comparisons with <150 SNPs are displayed. Dotted line denotes the 15 SNP cutoff used to define clonal relationship between isolates. **b**–**d** Heat maps of BSI patients’ zip code of residence for all isolates (panel **b**), or for isolates included in panel **a** (panels **c**, **d**). In panel **b**, areas denoted by dashed or gray lines are magnified in panels **c** and **d**, respectively. **e** Odds ratio comparting the characteristics between isolates included in panel **a** (“clustered) and all other isolates in the cohort (“non-clustered”). MA, methamphetamine.

We next determined the clonality of BSI isolates according to pairwise SNP distance. To standardize clonality analysis across our phylogenetically diverse cohort, we analyzed the 1782 genes shared by all genomes in our cohort (i.e., the ‘core genome’). By compiling the pairwise core genome SNP distances between index isolates and between surveillance isolates obtained from single individuals, we determined that isolates likely resulted from clonal transmission if their core genomes differed by <15 SNPs (Fig. 4a). Using this conservative cutoff, we identified 20 transmission clusters containing 46 index isolates, with all but one isolate being from IDU-BSI cases (Fig. 4b). These represented 29.2% (45/154) of all IDU-BSI isolates, but 78.6% (11/14) and 100% (3/3) of ST398/4163 and ST672 IDU-BSI isolates, respectively.

We subsequently investigated demographic and clinical features that could support an epidemiological link between cases within transmission clusters. Though patients in the entire cohort resided in 117 zip codes throughout Missouri and Illinois, 41/46 patients in transmission clusters shared or lived adjacent to the zip codes of other individuals within their cluster (Fig. 4c). The median time between index dates of cases within a cluster was 28 days (mean 95 days, range 1–355 days), compared to a median of 293 days (mean 393, range 1–1394 days) between cases in differing clusters (*p* = <0.0001). Fourteen of the 20 clusters (70%) demonstrated HCV seroconcordance between ≥2 individuals (Fig. 4b). Lastly, compared to cohort individuals not assigned to transmission clusters, patients in clusters were more likely to be under the age of 50 (*p* < 0.001), to experience prolonged bacteremia (*p* < 0.001), to be co-infected with HIV or HCV (*p* < 0.001), and to use injection opioids (*p* < 0.001) (Fig. 4d). Together these data describe multiple geographically and temporally localized *S. aureus* transmission networks, which share objective epidemiologic and biologic markers of needle sharing (i.e., report of IDU, HIV/HCV serology), exclusively among PWID.

### Secondary analysis reveals nonequivalent distribution of ST groups between cBSI and IDU-BSI isolates

**Fig. 5:**
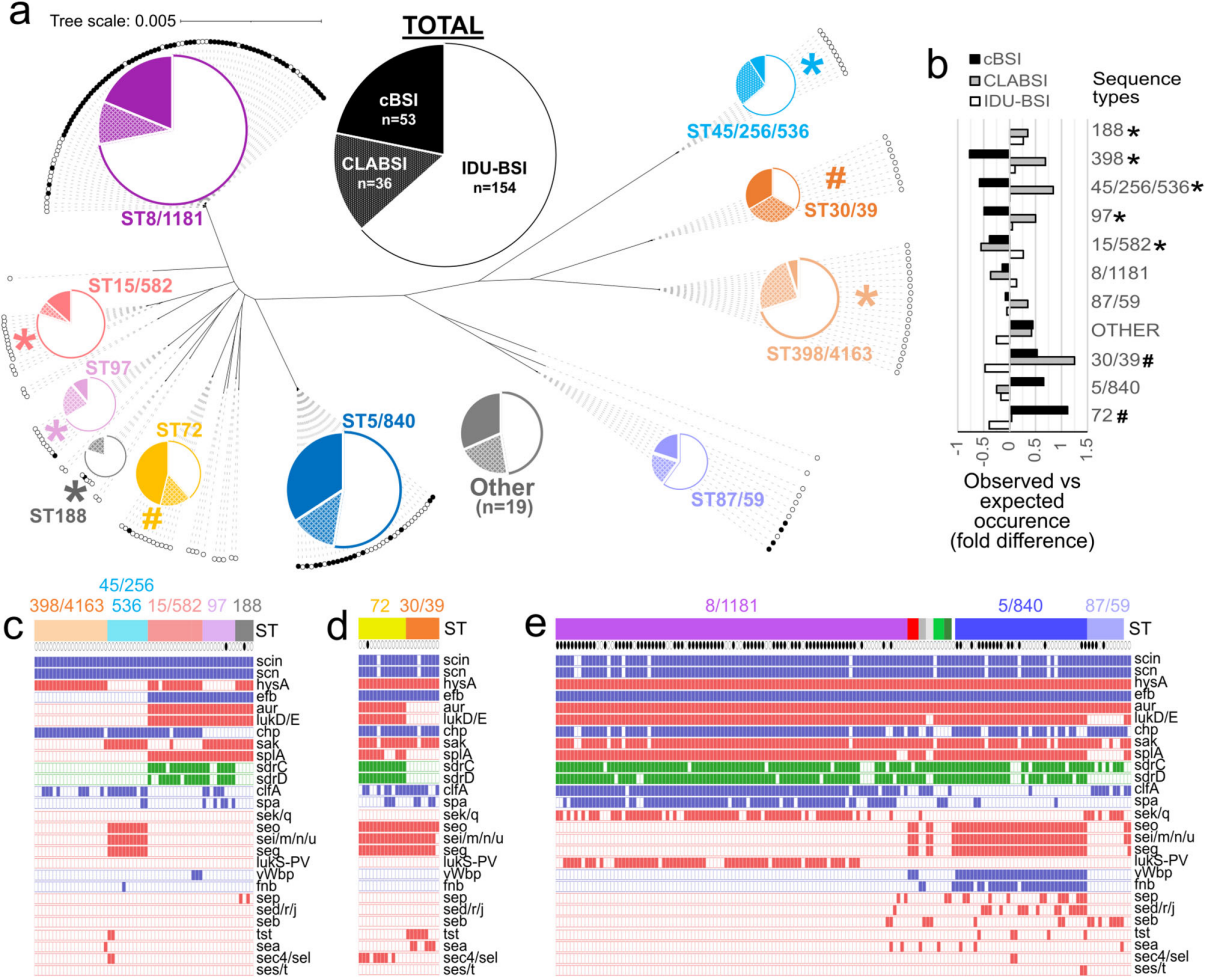
Differential distribution of phylogenetic subgroups and canonical virulence factor genes among different BSI types. **a** Unrooted version of the maximum-likelihood phylogenetic tree shown in Fig. 1, with each MRSA or MSSA isolate represented by a filled or empty circle, respectively. Pie charts represent the portion of each ST subgroup that was obtained for cBSI (solid), CLABSI (stippled) or IDU-BSI (white) cases. Only subgroups with ≥5 isolates are represented in pie charts. All other isolates (*n* = 19) were grouped as “Other”. **b** Fold differences of the observed versus expected incidence of each ST subgroup among cBSI (black bars), CLABSI (gray bars) and IDU-BSI (white bars) isolates in our cohort. No ST188 strains were identified among cBSI isolates. In panels **a** and **b**, (*) and (#) denote ST groups whose incidence was <75% of expected among cBSI and IDU-BSI isolates, respectively. **c**–**e** Representation of the genome content of ST subgroups underrepresented in cBSI (panel **c**), subgroups underrepresented in IDU-BSI (panel **d**) and all other subgroups (panel **e**). Each column represents a genome. First row denotes ST subgroup, with the genomes grouped as “Other” are lacking labels. Second row denotes MRSA (filled) or MSSA (empty) isolates. Subsequent rows show the presence (filled square) of canonical virulence factor genes, which are subclassified according to their roles in either immune evasion (blue), toxin production (red), or biofilm formation (green). Source data for this figure is derived from analysis of genome sequences uploaded at NCBI under BioProjects PRJNA694991 and PRJNA695316 and is available in tabular form in Supplementary Data 3.

As stated above, primary analysis revealed no difference in the distribution of ST groups between IDU-BSI to non-IDU BSI isolates. However, we noted a high incidence of CLABSI cases among the non-IDU cases in some lineages, such as ST398/4163 (Fig. 3). When non-IDU BSI isolates were sub-grouped into CLABSI and cBSI (*n* = 36 and *n* = 53, respectively), ST group distribution differed between cBSI and IDU-BSI cases (Supplementary Table 3, *p* = 0.035, Fisher exact test). When comparing observed to expected occurrence, underrepresented ST groups (i.e., ST groups observed at <75% of expected occurrence within a BSI subtype) were ST188, ST398/4163, ST45/256/536, ST97, and ST15/582 for cBSI, and ST30/39 and ST72 for IDU-BSI (Fig. 5a, b). These findings were consistent with differential selection of staphylococcal lineages according to BSI type.

### ST groups lacking multiple canonical virulence factors are underrepresented among cBSI isolates

Since genomic repertoire influences the differential ability of microbes to cause different disease forms, we evaluated for the presence of genomic antibiotic resistance and canonical staphylococcal virulence determinants in our cohort (Supplementary Data 3). As expected, gene distribution was principally according to ST group lineage, and the presence of resistance determinants correlated with lab susceptibility testing (Fig. 3 and Fig. 5c, d). The distribution of MRSA isolates did not differ between cBSI (20/53), CLABSI (8/36) and IDU-BSI isolates (64/154) (*p* = 0.094, Fisher exact test). Regarding the a priori selected list of fitness and virulence determinants (Supplementary Material 1), 100% of genomes contained the genes involved in iron acquisition and desiccation tolerance, and many genes involved in toxin production, immune evasion, and adhesion/biofilm formation were present in greater than 95% of isolates (Supplementary Table 1). For genes that occurred in less than 95% of total isolates, sequence types that were underrepresented in cBSI (Fig. 5c) lacked genes in all other sequences types (Fig. 5d, e), such as the staphylokinase and hyaloronidase genes *sak* and *hysA*, respectively. ST398/4163 isolates demonstrated the greatest paucity of canonical virulence factors (Fig. 5c).

## Discussion

To our knowledge, this is the first molecular epidemiological analysis comparing *S. aureus* isolates acquired from IDU-associated bloodstream infections (IDU-BSI) and conventional BSI (cBSI). Our case-control approach allowed us to identify characteristic features of IDU-BSI that are critical to consider in developing future investigations and interventions for this emerging disease.

Firstly, WGS revealed that person-to-person transmission of multiple MRSA and methicillin susceptible *S. aureus* (MSSA) lineages are fueling the epidemic of invasive infections among PWID in Missouri and Southern Illinois. The transmission of *S. aureus* within IDU networks had been previously proposed, but evidence was limited to studies with low-resolution molecular methods or small cohort sizes^10–13^. More recently, Packer et al.^14^ employed WGS to investigate MRSA colonization and disease isolates in PWID in Bristol, United Kingdom. They identified a single ST5 lineage predominated in their cohort, but their study was not designed to establish whether this phenomenon was exclusive to PWID, to identify transmission events, or to investigate dynamics of other lineages. By applying strict, empirically-derived clonality criteria comparable to those employed by other groups^41^, we found strong evidence of clonal expansion of pathogenic *S. aureus* IDU-BSI isolates, and that epidemiologically-linked BSI transmission clusters almost exclusively occurred among PWID. In multiple clusters, ≥3 IDU-BSI isolates shared a common ancestor, reflecting that staphylococcal IDU-BSI cases can result from community outbreaks, similar to HCV and HIV outbreaks linked to needle sharing practices among PWID^42^. Though we detected clonal expansion only among IDU-BSI cases, the relative underrepresentation (and, thus, reduced relative sequencing depth) of non-IDU BSI isolates in our genomic analysis could have decreased the likelihood of discovering clusters among non-IDU BSI cases, as well. However, the substantial difference in the proportion of matched IDU BSI and non-IDU BSI isolates belonging to transmission clusters (i.e., 29.2% [45/154] versus 1.1% [1/91]) firmly supports that clonal expansion is characteristic of the IDU-BSI epidemic.

Remarkably, some lineages (e.g., ST398/4163, ST672) were strongly associated with these IDU-BSI transmission clusters, suggesting that their pathogenicity depends heavily on IDU-associated risk factors. Many staphylococcal lineages underrepresented among cBSI cases lacked various canonical virulence factors associated with staphylococcal pathogenesis, suggesting a selective pressure favoring “well-equipped” lineages during conventional pathogenesis. Others have observed similar enrichment of virulence factors in invasive versus colonizing *S. aureus* isolates, with implicated virulence factors having a cumulative effect on invasiveness^43^. Conversely, direct inoculation of bacteria into the bloodstream and bypass of early stages of cBSI (e.g., abscess formation, mucosal invasion, early immune evasion, etc.), permits disease by “less virulent” lineages. The comparable-to-lower mortality of invasive Staphylococcal disease among PWID relative to disease in non-PWID popualtions^19,44^, despite the former’s association with prolonged bacteremia and often suboptimal antibiotic therapy^45,46^, may largely be due to the fact that the former occurs in a younger patient population. However, it is conceivable that the portion of IDU-BSI associated with lineages with reduced superantigen production or toxin secretion (both implicated in *S. aureus* sepsis) may also contribute to tempering IDU-associated infection severity. These implications, however, should not detract from the morbidity of IDU-BSI with “less virulent” lineages, as they were repeatedly implicated in BSI complications, including endocarditis, osteomyelitis, and septic arthritis in our cohort. Additional observational surveys of larger cohorts and preclinical research examining lineage-dependent pathogenesis, are required to confirm the impact of “less virulent” lineages in *S. aureus* invasive disease.

A notable example of a “less virulent” lineage underrepresented among cBSI cases was the clonal complex 398 (CC398), which includes ST398 and ST4163 (Supplementary Table 3). CC398 isolates in our cohort characteristically demonstrated macrolide resistance putatively mediated by *ermT* (Fig. 2), but they all lacked superantigens and many other canonical virulence factors. CC398 was first identified as a PVL-negative MRSA lineage in livestock in Europe, but was subsequently detected among MSSA colonizing diverse settings including community households and inmates in a jail holding tank^47–49^. Rarely implicated in human disease, CC398 was identified in only one of 81 MSSA soft tissue infections in a prior study in our area^50^. By contrast, CC398 was the most abundant exclusively MSSA lineage in our BSI cohort. CC398 was strongly associated with IDU transmission clusters, suggesting the lineage depends on intravenous inoculation in order to cause BSI. Indeed, though several non-IDU BSI cases were linked to CC398 in our cohort, they were almost exclusively CLABSI cases, where nosocomial bloodstream inoculation likely occurred. This demonstrates how preclinical models of IDU-BSI must account for the broader array of “less virulent” lineages that may be historically underrepresented among cBSI cases.

The dynamics mediating the high degree of relatedness among *S. aureus* IDU-BSI isolates may serve as a target for curbing the IDU-BSI epidemic and merits close examination. One explanation is that the intimate interactions characteristic to some PWID populations, such as congregation in non-traditional housing, sharing of drug use paraphernalia and transactional sexual exchanges, which may predispose to clonal expansion through sequential asymptomatic carriage within a PWID network. This, in turn, increases the likelihood that two individuals in a network develop IDU-BSI due to related *S. aureus* strains. However, this model presumes periods of genomic pool expansion during asymptomatic carriage punctuated by pool bottlenecking during colonization of new hosts and subsequent introduction into the bloodstream. It is unclear whether these population shifts would result in BSI cases with isolates displaying the high degree of genomic relatedness as what we observed between isolates subsequently obtained from a single BSI case and isolates within a single IDU-BSI transmission cluster (i.e., <15 core genome SNPs). An alternative explanation is subsequent infections resulting from sharing of drug preparation or delivery equipment colonized with *S. aureus*. Indeed, Kasper et al. previously found that 14% of cookers/filters used for injection of controlled-release opioids in a community were contaminated with *S. aureus* which could have been injected intravenously during routine substance use^9^. In either scenario, socioeconomic status factors such as homelessness and the low accessibility of needle exchange services in Missouri (which is a needle non-exchange state) may have contributed to the relatively high prevalence of transmitted isolates in the current study.

A third explanation for the clonal nature of IDU-BSI isolates would be bloodborne transmission. We confirmed IDU-BSI was associated with prolonged durations of bacteremia and frequent AMA discharges. So, although *S. aureus* invasive infections are generally considered to have acute and fulminant presentations, these infections conceivably take a more chronic course in a) individuals receiving intermittent, abbreviated antimicrobial therapy resulting in a “lower-grade” bacteremia or b) BSI patients with incompletely treated, secondary infections (e.g., endocarditis, osteomyelitis, occult abscesses, etc.) that can serve as chronic sources for recurrent bacteremia. Indeed, our cohort included an individual who had clonal *S. aureus* isolates obtained from blood specimens over a seven month period, without intervening negative blood cultures. Furthermore, seven transmission clusters contained IDU-BSI patients from whom clonal *S. aureus* isolates were obtained from at least two separate hospitalizations. Thus, IDU-BSI patients who defer medical attention could plausibly serve as reservoirs for bloodborne transmission through sharing of needles or drug preparation equipment. This provocative scenario requires further investigation, as it would shift priority towards clearance of bacteremia as a means to curb IDU-BSI propagation and inform the approach for assessing risk factors for *S. aureus* BSI in this population.

Our investigation was limited to a single region where needle exchanges are prohibited, thus, limiting the generalizability of our findings. However, this represents a unique opportunity to examine the indirect impact of social IDU mitigation strategies practiced in other states on the BSI epidemic. Because the validity of contact tracing is complicated by recall limitations and socioeconomic instability often experienced by PWID, we were unable to validate our genomically-derived transmission clusters with retrospective contact tracing. However, our conservative SNP threshold for case clustering was deliberately chosen to minimize false-positive effects. Since our WGS cohort did not include isolates from non-endovascular host sites, we could not investigate whether asymptomatic colonization is a prerequisite for BSI among PWID. Further, the impact of different inpatient interventions such as ICU admission or cardiac surgery which could affect mortality differences between PWID and non-PWID groups was not assessed in this study. Lastly, our follow-up was limited to data present in the electronic medical record and out of hospital deaths which could contribute to mortality differences would not be captured in this study. Despite these limitations, the comparative design of our study combined with robust clinical and genomic data provides valuable insight into the epidemiological and pathophysiological impact of the propagation of multiple *S. aureus* lineages among PWID.

## Conclusion

We identified clonal expansion of multiple *S. aureus* lineages among IDU-BSI isolates, but not non-IDU BSI isolates, in a community with limited access to needle exchange facilities. In the setting of expanding numbers of staphylococcal IDU-BSI cases consideration should be given to treating IDU-associated invasive staphylococcal infections as a communicable disease.

### Reporting summary

Further information on research design is available in the Nature Research Reporting Summary linked to this article.

## Supplementary information

Description of Additional Supplementary Files

Supplemental Material

Supplementary Data 1

Supplementary Data 2

Supplementary Data 3

Reporting Summary

## Data Availability

The data that support the findings of this study are available within the paper and its supplementary information files. The source data underlying Figs. 3, 4 & 5 are derived from whole-genome sequencing of the strains in this project. Whole-genome sequence read files are uploaded to NCBI under BioProjects PRJNA694991 and PRJNA695316. Source metadata for Fig. 3 & 5 can be accessed as Supplementary Data 2. Source Data for Fig. 4a can be accessed as Supplementary Data 3. The remaining source data on individual patient outcomes and locations used in Figs. 2 and 4b–e cannot be provided because it contains elements of protected health information and the ethical approval does not cover placing individual patient level data into publicly open repositories. Relevant portions of those data can be accessed from the authors upon relevant ethical approval by contacting the corresponding author on reasonable request.

## References

[1] Golden MR (2019). Outbreak of human immunodeficiency virus infection among heterosexual persons who are living homeless and inject drugs—Seattle, Washington, 2018. MMWR. Morbidity Mortality Weekly Rep..

[2] Ramachandran S (2018). A large HCV transmission network enabled a fast-growing HIV outbreak in rural Indiana, 2015. EBioMedicine.

[3] Hartnett KP (2019). Bacterial and fungal infections in persons who inject drugs—Western New York, 2017. MMWR. Morbidity Mortality Weekly Report.

[4] See I (2020). National public health burden estimates of endocarditis and skin and soft-tissue infections related to injection drug use: a review. J. Infect. Dis..

[5] Jackson KA (2018). Invasive methicillin-resistant Staphylococcus aureus infections among persons who inject drugs - six sites, 2005–2016. MMWR. Morbidity Mortality Weekly Rep..

[6] McCarthy NL (2020). Bacterial Infections Associated With Substance Use Disorders, Large Cohort of United States Hospitals, 2012–2017. Clin. Infect. Dis..

[7] Mermel LA (2009). Clinical practice guidelines for the diagnosis and management of intravascular catheter-related infection: 2009 Update by the Infectious Diseases Society of America. Clin. Infect. Dis..

[8] O’Grady NP (2011). Guidelines for the prevention of intravascular catheter-related infections. Am. J. Infect. Control.

[9] Kasper KJ (2019). A controlled-release oral opioid supports S. aureus survival in injection drug preparation equipment and may increase bacteremia and endocarditis risk. PloS ONE.

[10] Levine DP, Crane LR, Zervos MJ (1986). Bacteremia in narcotic addicts at the Detroit Medical Center. II. Infectious endocarditis: a prospective comparative study. Rev. Infect. Dis..

[11] Craven DE, Rixinger AI, Goularte TA, McCabe WR (1986). Methicillin-resistant Staphylococcus aureus bacteremia linked to intravenous drug abusers using a “shooting gallery”. Am. J. Med..

[12] Quagliarello B (2002). Strains of Staphylococcus aureus obtained from drug-use networks are closely linked. Clin. Infect. Dis..

[13] Gilbert M (2006). Outbreak in Alberta of community-acquired (USA300) methicillin-resistant Staphylococcus aureus in people with a history of drug use, homelessness or incarceration. Canadian Med. Assoc. J..

[14] Packer, S. et al. Clonal expansion of community-associated meticillin-resistant Staphylococcus aureus (MRSA) in people who inject drugs (PWID): prevalence, risk factors and molecular epidemiology, Bristol, United Kingdom, 2012 to 2017. *Euro surveillance: bulletin Europeen sur les maladies transmissibles = European communicable disease bulletin* **24**, 10.2807/1560-7917.Es.2019.24.13.1800124 (2019). 10.2807/1560-7917.ES.2019.24.13.1800124 PMC6446509 30940316

[15] Arshad, S. et al. IV Drug abuse in methicillin-resistant *Staphylococcus aureus* (MRSA) bacteremia: epidemiology, strain characteristics and outcomes Presented at IDWeek; San Diego, CA. (2012).

[16] Halavaara, M., Martelius, T., Anttila, V.-J. & Järvinen, A. Three separate clinical entities of infective endocarditis—a population-based study from Southern Finland 2013–2017. *Open Forum Infect. Dis.* **7**10.1093/ofid/ofaa334 (2020). 10.1093/ofid/ofaa334 PMC7473740 32913877

[17] Fowler VG (2005). Staphylococcus aureus EndocarditisA consequence of medical progress. JAMA.

[18] Asgeirsson, H., Thalme, A. & Weiland, O. Low mortality but increasing incidence of Staphylococcus aureus endocarditis in people who inject drugs: experience from a Swedish referral hospital. *Medicine* **95**, e5167 (2016). 10.1097/MD.0000000000005617 PMC5266062 27930590

[19] Appa, A. et al. Comparative one-year outcomes of invasive Staphylococcus aureus infections among persons with and without drug use: an observational cohort study. *Clin. Infect. Dis.*10.1093/cid/ciab367 (2021). 10.1093/cid/ciab367 PMC8800187 33904900

[20] Tong SY, Davis JS, Eichenberger E, Holland TL, Fowler VG (2015). Staphylococcus aureus infections: epidemiology, pathophysiology, clinical manifestations, and management. Clin. Microbiol. Rev..

[21] Marks LR (2020). A comparison of medication for opioid use disorder treatment strategies for persons who inject drugs with invasive bacterial and fungal infections. J. Infect. Dis..

[22] Kuehl, R. et al. Defining persistent Staphylococcus aureus bacteraemia: secondary analysis of a prospective cohort study. *Lancet Infect. Dis.*10.1016/S1473-3099(20)30447-3 (2020). 10.1016/S1473-3099(20)30447-3 32763194

[23] Elixhauser A, Steiner C, Harris DR, Coffey RM (1998). Comorbidity measures for use with administrative data. Med. Care.

[24] Clinical and Laboratory Standards Institute. *Performance Standards for Antimicrobial Susceptibility Testing* 27th edn. CLSI document M100-S27. (Clinical and Laboratory Standards Institute, 2017).

[25] Potter, R. F. et al. Population Structure, Antibiotic Resistance, and Uropathogenicity of Klebsiella variicola. *MBio* **9**10.1128/mBio.02481-18 (2018). 10.1128/mBio.02481-18 PMC6299229 30563902

[26] Bolger AM, Lohse M, Usadel B (2014). Trimmomatic: a flexible trimmer for Illumina sequence data. Bioinformatics.

[27] Wick RR, Judd LM, Gorrie CL, Holt KE (2017). Unicycler: Resolving bacterial genome assemblies from short and long sequencing reads. PLoS Comput. Biol..

[28] Gurevich A, Saveliev V, Vyahhi N, Tesler G (2013). QUAST: quality assessment tool for genome assemblies. Bioinformatics.

[29] Pritchard L, Glover RH, Humphris S, Elphinstone JG, Toth IK (2016). Genomics and taxonomy in diagnostics for food security: soft-rotting enterobacterial plant pathogens. Anal. Methods.

[30] Bortolaia V (2020). ResFinder 4.0 for predictions of phenotypes from genotypes. J. Antimicrob. Chemother..

[31] Page AJ, Taylor B, Keane JA (2016). Multilocus sequence typing by blast from de novo assemblies against PubMLST. J. Open Source Softw..

[32] Seemann T (2014). Prokka: rapid prokaryotic genome annotation. Bioinformatics.

[33] Manara S (2018). Whole-genome epidemiology, characterisation, and phylogenetic reconstruction of Staphylococcus aureus strains in a paediatric hospital. Genome Med..

[34] Diep BA, Carleton HA, Chang RF, Sensabaugh GF, Perdreau-Remington F (2006). Roles of 34 virulence genes in the evolution of hospital- and community-associated strains of methicillin-resistant Staphylococcus aureus. J. Infect. Dis..

[35] Page AJ (2015). Roary: rapid large-scale prokaryote pan genome analysis. Bioinformatics.

[36] Stamatakis A (2014). RAxML version 8: a tool for phylogenetic analysis and post-analysis of large phylogenies. Bioinformatics.

[37] Letunic I, Bork P (2016). Interactive tree of life (iTOL) v3: an online tool for the display and annotation of phylogenetic and other trees. Nucleic Acids Res..

[38] Page AJ (2016). SNP-sites: rapid efficient extraction of SNPs from multi-FASTA alignments. Microb. Genom..

[39] Shannon P (2003). Cytoscape: a software environment for integrated models of biomolecular interaction networks. Genome Res..

[40] Team, R. C. *R.: A Language and Environment for Statistical Computing* (*R Foundation for Statistical Computing*, 2020).

[41] Goyal M (2019). Genomic evolution of Staphylococcus aureus during artificial and natural colonization of the human nose. Fron. Microbiol..

[42] Prevention, C. f. D. C. a. 2019: Illuminating HIV outbreaks with AMD. (2019).

[43] Peacock SJ (2002). Virulent combinations of adhesin and toxin genes in natural populations of *Staphylococcus aureus*. Infect. Immunity.

[44] Fowler VG (2005). Staphylococcus aureus endocarditis: a consequence of medical progress. JAMA.

[45] Rudasill SE (2019). Clinical outcomes of infective endocarditis in injection drug users. J. Am. College Cardiol..

[46] Kimmel, S. D. et al. Against medical advice discharges in injection and non-injection drug use-associated infective endocarditis: a nationwide cohort study. *Clin. Infect. Dis.*10.1093/cid/ciaa1126 (2020). 10.1093/cid/ciaa1126 PMC8563193 32756935

[47] Smith TC, Wardyn SE (2015). Human infections with Staphylococcus aureus CC398. Curr. Environ. Health Rep..

[48] Uhlemann A-C (2017). Evolutionary dynamics of pandemic methicillin-sensitive—Staphylococcus aureus—ST398 and its international spread via routes of human migration. mBio.

[49] David MZ (2013). Asymptomatic carriage of sequence type 398, spa type t571 Methicillin—susceptible *Staphylococcus aureus* in an Urban Jail: a newly emerging, transmissible pathogenic strain. J. Clin. Microbiol..

[50] Orscheln RC (2009). Contribution of genetically restricted, methicillin-susceptible strains to the ongoing epidemic of community-acquired Staphylococcus aureus infections. Clin. Infect. Dis..

