# Supplementary material for: Staphylococcus aureus injection drug use-associated bloodstream infections are propagated by community outbreaks of diverse lineages
Source: Commun Med (Lond). 2021 Nov 30;1:52. doi: 10.1038/s43856-021-00053-9 (PMC9053277; doi:10.1038/s43856-021-00053-9)
Supplement: Supplementary file 1 — Description of Additional Supplementary Files [file 43856_2021_53_MOESM1_ESM.pdf]

Description of Additional Supplementary Files

File Name: Supplementary Data 1

Description: List and nucleotide sequences of putative virulence genes selected *a priori* for genomic analysis

File Name: Supplementary Data 2

Description: Antimicrobial susceptibility and genomic data.

File Name: Supplementary Data 3

Description: Antimicrobial susceptibility and genomic data.
