# Supplementary material for: Staphylococcus aureus injection drug use-associated bloodstream infections are propagated by community outbreaks of diverse lineages
Source: Commun Med (Lond). 2021 Nov 30;1:52. doi: 10.1038/s43856-021-00053-9 (PMC9053277; doi:10.1038/s43856-021-00053-9)
Supplement: Supplementary file 2 — Supplemental Material [file 43856_2021_53_MOESM2_ESM.pdf]

Staphylococcus aureus injection drug use-associated bloodstream infections are propagated by community outbreaks of diverse lineages

Laura R. Marks[1*], Juan J. Calix[1,2*], John A. Wildenthal[1], Meghan A. Wallace[3], Sanjam Sawhney[2], Eric M. Ransom[3], Michael J. Durkin[1], Jeffrey P Henderson[1,4,5], Carey-Ann D. Burnham[3,4#], and Gautam Dantas[2,3,4,5,#]

Affiliations:

[1]Division of Infectious Diseases, Washington University in St. Louis School of Medicine. St. Louis, Missouri, United States of America

[2] The Edison Family Center for Genome Sciences and Systems Biology, Washington University School of Medicine, St. Louis, Missouri, United States of America

[3]Department of Pathology and Immunology, Washington University School of Medicine, St. Louis, Missouri, United States of America

[4] Department of Molecular Microbiology, Washington University School of Medicine, St. Louis, Missouri, United States of America

[5]Department of Biomedical Engineering, Washington University, St. Louis, Missouri, United States of America

* These authors contributed equally: L.R.M, J.J.C

# Corresponding authors: C-A.D.B. (cburnham@wustl.edu); and G.D. (dantas@wustl.edu)

Supplementary Table 1: Demographics and Clinical Characteristics of all *S. aureus* Blood-stream Isolates at Barnes Jewish Hospital from 1/2016 – 12/2019 by Injection Drug Use Status

	Non-IDU BSI Cases	IDU-BSI Cases	FDR corrected p value
	N=1261 (%)	*N= 173 (%)*	
**Demographics**			
Age (mean years, SD)	55±15	39±10	<0.001
Female[a]	473 (37.5)	77 (44.5)	0.371
Discharge AMA	18 (1.4)	40 (23.1)	<0.001
**Coinfections**			
Hepatitis C Virus Infection	101 (8.0)	119 (68.7)	<0.001
HIV infection	20 (1.6)	10 (5.8)	0.055
**Elixhauser comorbidities**			
No. Elixhauser Comorbidities (Mean, SD)	8.9 (3.6)	8.8 (3.7)	0.499
AIDS and HIV	20 (1.6)	10 (5.8)	0.011
Alcohol Abuse	134 (10.6)	26 (15.0)	0.148
Rheumatoid Arthritis, Collagen Vascular Disease	119 (9.4)	15 (8.7)	0.772
Blood loss anemia	82 (6.5)	10 (5.8)	0.775
Congestive heart failure	536 (42.5)	51 (29.5)	0.002
Coagulopathy	605 (48)	81)	0.775
Chronic Pulmonary Disorder	451 (35.8)	64 (37.0)	0.771
Drug Abuse	169 (13.4)	149 (86.1)	0.415
Depression	506 (40.1)	72 (41.6)	0.768
Diabetes, uncomplicated	412 (32.7)	23 (13.3)	<0.001
Diabetes, complicated	538 (42.7)	18 (10.4)	<0.001
Fluid and electrolyte disorder	1061 (84.1)	143 (82.7)	0.751
Hypertension	995 (78.9)	86 (49.7)	<0.001
Hypothyroidism	227 (18.0)	10 (5.8)	<0.001
Liver disease	308 (24.4)	71 (41.0)	<0.001
Lymphoma	96 (7.6)	2 (1.2)	<0.001
Metastatic Cancer	162 (12.8)	6 (3.5)	<0.001
Other Neurologic Disorders	441 (35.0)	51 (29.5)	0.216
Obesity	447 (35.4)	21 (12.1)	<0.001
Paralysis	227 (18.0)	27 (15.6)	0.546
Peripheral Vascular Disorders	487 (38.6)	83 (48.0)	0.031
Pulmonary Circulation Disorders	204 (16.2)	92 (53.2)	<0.001
Psychoses	129 (10.2)	38 (22.0)	<0.001
Renal Failure	576 (45.7)	55 (31.8)	<0.001
Solid Tumor, without metastases	234 (18.6)	8 (4.6)	<0.001
Weight Loss	601 (47.7)	59 (34.1)	0.001
***S. aureus* Clinical Infection Characteristics and Outcomes**			
Infective endocarditis	400 (32.8)	106 (58.4)	<0.001

Osteomyelitis	261 (20.7)	34 (19.6)	0.249
Septic Arthritis	86 (6.8)	27 (15.6)	<0.001
Necrotizing SSTI	36 (2.8)	24 (13.9)	<0.001
CLABSI	84 (6.7)	1 (0.6)	0.004
Duration of Bacteremia (mean days, SE)	2.5±0.3	3.8±0.4	<0.001
Prolonged Bacteremia (>5 days)	148 (11.7)	53 (30.6)	<0.001
Methicillin Resistance	743 (58.9)	97 (56.1)	0.728
1-year Mortality	409 (32.4)	27 (15.6)	<0.001

[a]Unless otherwise specified, all values are n (%)

AMA, against medical advice; CLABSI, central line-associated bacteremia; HIV, human immunodeficiency virus; IDU, injection drug use; MRSA, methicillin resistant Staphylococcus aureus; PWID, person who injects drugs; SD standard deviation; SE, standard error; SSTI, Skin/soft tissue infection.

Supplementary Table 2: Factors Associated with 1-year Mortality for *S. aureus* Blood-stream Infections

	Univariate Analysis		Multivariable Analysis	
**Variable**	Hazard Ratio (95% CI)	P value	Hazard Ratio (95% CI)	P value
**Demographics**				
**Age**	1.020 (1.01 - 1.03)	<0.001	1.029 (1.02 - 1.04)	<0.001
**Female**	1.21 (0.99 – 1.47)	0.051	1.32 (1.02 - 1.68)	0.021
**S. aureus Clinical Infection Characteristics and Outcomes**				
**Methicillin Resistant Staphylococcus aureus**	1.197 (0.97 - 1.43)	0.105		
**Bacteremia 5+ Days**	0.99 (0.73 - 1.34)	0.951		
**Endocarditis**	1.27 (0.89 - 1.79)	0.176		
**Injection Drug Use Associated**	0.63 (0.40 - 0.98)	0.047	0.78 (0.62 - 0.98)	0.04
**Elixhauser comorbidities**				
**AIDS and HIV**	0.83 (0.32 - 2.17)	0.706		
**Alcohol Abuse**	1.16 (0.78 - 1.74)	0.465		
**Blood loss anemia**	1.25 (0.88 - 1.78)	0.221		
**Coagulopathy**	1.53 (1.23 – 1.91)	<0.001	1.63 (1.24 - 2.15)	<0.001
**Congestive heart failure**	1.21 (0.97 – 1.49)	0.086	1.03 (0.77 - 1.35)	0.011
**Chronic Pulmonary Disorder**	1.15 (0.088 - 1.49)	0.309		
**Depression**	0.78 (0.59 - 1.01)	0.057	0.80 (0.63 - 1.03)	0.085
**Diabetes, uncomplicated**	1.01 (0.73 - 1.39)	0.942		
**Diabetes, complicated**	0.94 (0.68 - 1.29)	0.700		
**Drug Abuse**	0.68 (0.56 – 0.88)	0.002	*not included, duplicates PWID data-point	
**Hypertension**	1.08 (0.77 - 1.50)	0.677		
**Hypothyroidism**	1.45 (1.05 - 1.98)	0.023	1.25 (0.88 - 1.74)	0.204
**Fluid and electrolyte disorder**	3.02 (1.94 – 4.73)	<0.001	2.50 (1.65 - 3.79)	<0.001
**Lymphoma**	0.90 (0.62 - 1.30)	0.578		
**Liver disease**	1.14 (0.85 - 1.52)	0.390		
**Metastatic Cancer**	1.32 (0.93 - 1.88)	0.126		
**Other Neurologic Disorders**	1.32 (0.93 - 1.88)	0.126		
**Obesity**	0.84 (0.64 - 1.10)	0.209		
**Paralysis**	1.21 (0.88 - 1.65)	0.244		
**Peripheral Vascular Disorders**	1.10 (0.85 - 1.43)	0.471		
**Pulmonary Circulation Disorders**	0.831 (0.60 - 1.15)	0.264		
**Psychoses**	0.62 (0.48 – 0.93)	0.021	0.45 (0.29 - 0.69)	<0.001
**Renal Failure**	1.03 (0.83 - 1.28)	0.786		
**Solid Tumor, without metastases**	1.33 (1.01 – 1.71)	0.061	1.78 (1.31 - 2.39)	<0.001
**Weight Loss**	1.22 (0.98 – 1.50)	0.069	1.37 (1.08 - 1.75)	0.010

Supplementary Table 3: ***S. aureus*** **multilocus Sequence type group composition according to Blood-stream Infection subtypes**

	**IDU-BSI**	**non-IDU BSI**		**Total**	**OR [CI95%]**			
		CLABSI	cBSI		IDU-BSI vs other	CLABSI vs other	cBSI vs other	IDU-BSI vs cBSI
**ST8/1181**	69	9	18	96	1.86 [1.03-3.38]	0.46 [0.18-1.07]	0.74 [0.37-1.45]	1.57 [0.79-3.23]
**ST5/840**	19	4	13	36	0.60 [0.27-1.31]	0.68 [0.16-2.13]	2.35 [1.00-5.34]	0.44 [0.18-1.05]
**ST398/4163**	14	5	1	20	1.38 [0.48-4.56]	2.06 [0.55-6.52]	0.14 [0.003-0.91]	5.17 [0.75-223.83]
**ST15/582**	12	1	2	15	2.41 [0.63-13.71]	0.39 [0.01-2.76]	0.54 [0.06-2.48]	2.15 [0.45-20.42]
**ST72**	5	2	6	13	0.34 [0.08-1.23]	1.05 [0.11-5.13]	3.32 [0.88-3.32]	0.27 [0.06-1.09]
**ST45/256/536**	7	3	1	11	1.01 [0.25-4.85]	2.25 [0.37-10.01]	0.35 [0.01-2.45]	2.47 [0.31-113.66]
**ST87/59**	6	2	2	10	0.86 [0.20-4.27]	1.46 [0.15-7.77]	0.71 [0.07-3.47]	1.03 [0.18-10.79]
**ST30/39**	3	3	3	9	0.28 [0.04-1.33]	3.03 [0.47-15.01]	1.83 [0.29--8.96]	0.33 [0.04-2.57]
**ST97**	6	2	1	9	1.16 [0.24-7.36]	1.68 [0.16-9.32]	0.91 [0.02-7.29]	2.10 [0.25-98.77]
**ST188**	4	1	0	5	2.34 [0.23-116.80]	1.45 [0.03-15.19]	N/A	N/A
**OTHER[1]**	9	4	6	19	0.49 [0.17-1.41]	1.59 [0.36-5.45]	1.73 [0.51-5.21]	0.49 [0.15-1.76]
**TOTAL**	**154**	**36**	**53**	**243**				

[1]Includes singleton isolates and STs 672, 6, 20, 27, 1 and 1159

CLABSI, central line-associated bacteremia; cBSI, conventional blood-stream infection; IDU-BSI, injection drug use associated blood-stream infection;
