# Supplementary material for: Staphylococcus aureus injection drug use-associated bloodstream infections are propagated by community outbreaks of diverse lineages
Source: Commun Med (Lond). 2021 Nov 30;1:52. doi: 10.1038/s43856-021-00053-9 (PMC9053277; doi:10.1038/s43856-021-00053-9)
Supplement: Supplementary file 6 — Reporting Summary [file 43856_2021_53_MOESM6_ESM.pdf]

nature research

Corresponding author(s): Laura Marks

Last updated by author(s): 10/30/2021

Reporting Summary

Nature Research wishes to improve the reproducibility of the work that we publish. This form provides structure for consistency and transparency in reporting. For further information on Nature Research policies, see our Editorial Policies and the Editorial Policy Checklist.

Statistics

For all statistical analyses, confirm that the following items are present in the figure legend, table legend, main text, or Methods section.

n/a	Confirmed	
☐	☒	The exact sample size (n) for each experimental group/condition, given as a discrete number and unit of measurement
☐	☒	A statement on whether measurements were taken from distinct samples or whether the same sample was measured repeatedly
☐	☒	The statistical test(s) used AND whether they are one- or two-sided<br>*Only common tests should be described solely by name; describe more complex techniques in the Methods section.*
☐	☒	A description of all covariates tested
☐	☒	A description of any assumptions or corrections, such as tests of normality and adjustment for multiple comparisons
☐	☒	A full description of the statistical parameters including central tendency (e.g. means) or other basic estimates (e.g. regression coefficient) AND variation (e.g. standard deviation) or associated estimates of uncertainty (e.g. confidence intervals)
☐	☒	For null hypothesis testing, the test statistic (e.g. F, t, r) with confidence intervals, effect sizes, degrees of freedom and P value noted<br>*Give P values as exact values whenever suitable.*
☒	☐	For Bayesian analysis, information on the choice of priors and Markov chain Monte Carlo settings
☒	☐	For hierarchical and complex designs, identification of the appropriate level for tests and full reporting of outcomes
☒	☐	Estimates of effect sizes (e.g. Cohen's d, Pearson's r), indicating how they were calculated

Our web collection on statistics for biologists contains articles on many of the points above.

Software and code

Policy information about availability of computer code

Data collection	Data used in this study was from individual patient samples. Whole genome sequence read files are uploaded to NCBI under BioProjects PRJNA694991 and PRJNA695316.
Data analysis	All software used for data analysis is described in the methods section and is commercially available or open source. Software used includes R, ResFinder v 4.0, Prokka v 1.13.7, Roary v3.13, raxML v 8.2.11, iTOL and Cytoscape v4.0 R,

For manuscripts utilizing custom algorithms or software that are central to the research but not yet described in published literature, software must be made available to editors and reviewers. We strongly encourage code deposition in a community repository (e.g. GitHub). See the Nature Research guidelines for submitting code & software for further information.

Data

Policy information about availability of data

All manuscripts must include a data availability statement. This statement should provide the following information, where applicable:

- Accession codes, unique identifiers, or web links for publicly available datasets
- A list of figures that have associated raw data
- A description of any restrictions on data availability

Whole-genome sequence read files are uploaded to NCBI under BioProjects PRJNA694991 and PRJNA695316. Additional raw data used for the analyses in this study is provided as supplemental data.

Field-specific reporting

Please select the one below that is the best fit for your research. If you are not sure, read the appropriate sections before making your selection.

☒ Life sciences ☐ Behavioural & social sciences ☐ Ecological, evolutionary & environmental sciences

For a reference copy of the document with all sections, see nature.com/documents/nr-reporting-summary-flat.pdf

Life sciences study design

All studies must disclose on these points even when the disclosure is negative.

Sample size	Sample size included all injection drug use associated S. aureus isolates which occurred during the study time-period (time-period determined by outbreak dynamics) and met quality thresholds for genomic sequencing.
Data exclusions	There were no data exclusions.
Replication	No replication was performed due to the nature of the genomic epidemiology study.
Randomization	This was a retrospective analysis of a case-control cohort. No randomization was performed. Covariate including patient characteristics (type of substance used, cotransmitted diseases) are adjusted for in the network analysis described in the methods section.
Blinding	This was a retrospective analysis of a case-control cohort. Investigators were not blinded to group assignment for individual staphylococcal genomes.

Reporting for specific materials, systems and methods

We require information from authors about some types of materials, experimental systems and methods used in many studies. Here, indicate whether each material, system or method listed is relevant to your study. If you are not sure if a list item applies to your research, read the appropriate section before selecting a response.

Materials & experimental systems

n/a	Involved in the study	
☒	☐	Antibodies
☒	☐	Eukaryotic cell lines
☒	☐	Palaeontology and archaeology
☒	☐	Animals and other organisms
☒	☐	Human research participants
☐	☒	Clinical data
☒	☐	Dual use research of concern

Methods

n/a	Involved in the study	
☒	☐	ChIP-seq
☒	☐	Flow cytometry
☒	☐	MRI-based neuroimaging

Clinical data

Policy information about clinical studies

All manuscripts should comply with the ICMJE guidelines for publication of clinical research and a completed CONSORT checklist must be included with all submissions.

Clinical trial registration	N/a - This was not a clinical trial.
Study protocol	N/a
Data collection	Clinical data collection occurred for staphylococcus aureus bloodstream isolates cultured at Barnes-Jewish Hospital (BJH) between 1/2016-12/2019. Cases were manually reviewed by an infectious diseases physician and patient demographics, drug use history, clinical course and clinical microbiological data were paired with genomics data from whole genome sequencing. The study was issued a waiver of consent by the Washington University IRB. (IRB# 201804183, 201907187, 201911072, 202007171).
Outcomes	N/a
